# Stability, Microstructure, and Rheological Properties of CaCO_3_ S/O/W Calcium-Lipid Emulsions

**DOI:** 10.3390/foods10092216

**Published:** 2021-09-18

**Authors:** Jie Zhang, Gongwei Li, Duoxia Xu, Yanping Cao

**Affiliations:** Beijing Advanced Innovation Center for Food Nutrition and Human Health (BTBU), School of Food and Health, Beijing Engineering and Technology Research Center of Food Additives, Beijing Higher Institution Engineering Research Center of Food Additives and Ingredients, Beijing Key Laboratory of Flavor Chemistry, Beijing Laboratory for Food Quality and Safety, Beijing Technology & Business University, Beijing 100048, China; zjzxw722@163.com (J.Z.); ligwbtbuer@163.com (G.L.); xdxbtbu@126.com (D.X.)

**Keywords:** S/O/W emulsions, CaCO_3_, physicochemical stability, rheology, microstructure

## Abstract

Calcium carbonate (CaCO_3_) is a commonly used fortified calcium, but poor suspension stability and easy precipitation seriously limited its food processing and products application. The formation of CaCO_3_ loaded microparticles based on the form of solid/oil/water (S/O/W) emulsion is a promising method to improve the dispersion stability of CaCO_3_ in liquid food. In this study, CaCO_3_, soybean oil, and sodium caseinate (NaCas) were used as the solid, oil, and W phase, respectively. The fabrication involved two steps: the S/O emulsion was prepared by adding CaCO_3_ into soybean oil by magnetic stirring and high-speed shearing, and then put the S/O crude emulsion into NaCas solution (W phase) to obtain S/O/W emulsion by high-speed blender. The particle size distribution, zeta potential, stability of the microsphere, infrared spectral analysis, and XRD of the S/O/W calcium-lipid microsphere were explored. The stability and rheological mechanism of S/O/W calcium-lipid emulsion were investigated by combining the microstructure, shear rheological, and microrheological properties. It was found that the emulsion particles have more uniform particle size distribution and no aggregation, and the stability of the emulsion was improved with increasing the content of NaCas. The mean square displacement (MSD) curve and solid-liquid equilibrium (SLB) value of S/O/W emulsion increased with the increase in NaCas concentration, and the viscosity behavior is dominant. The results of confocal laser microscopy (CLSM) and cryo-scanning electron microscopy (Cryo-SEM) showed that the three-dimensional network structure of S/O/W emulsions was more compact, and the embedding effect of calcium carbonate (CaCO_3_) was slightly improved with the increase in NaCas concentration. According to infrared spectrum and XDR analysis, the addition of CaCO_3_ into the emulsion system caused crystal structure distortion. This study provides a reference for solving the dispersibility of insoluble calcium salt in liquid food.

## 1. Introduction

The addition of functional nutrients to the food system is considered to be a simple and effective nutritional supplement method to reduce and prevent various chronic diseases [1,2,3,4]. Mineral elements such as calcium is an essential mineral for human nutrition, with the currently recommended dietary allowance being 1000 mg daily for adults [5]. Calcium carbonate (CaCO_3_), citrate, gluconate, and lactate are forms of calcium used in dietary supplements [6]. Due to the highest percentage of elemental calcium (40% *w*/*w*), low cost, and chemical stability, CaCO_3_ is the most commonly used form of calcium supplement in the market [7]. While, CaCO_3_ has problems of poor solubility and low digestion and absorption, which seriously limit their application in food processing and products [8]. The main problem of dairy products with insoluble calcium salt is that insoluble calcium salt is easy to precipitate during storage. In actual production, the insoluble calcium salt is directly mixed with dairy products in the form of micro-powdered calcium particles. Therefore, it is needed to develop new nutrient delivery system carriers of CaCO_3_ to solve the dispersion stability of insoluble calcium salt in liquid food.

At present, the commonly used carriers of food delivery systems mainly include microgel, nanoemulsion, emulsion, microemulsion, solid-phase lipid particles, multiple emulsion, liposome, etc. It can be used for steady-state and controlled release of fat-soluble and water-soluble nutrients, effectively improving the stability and digestion and absorption rate of nutrients. However, there were few reports on carriers of solid-phase components such as mineral elements. To realize the homogenization of solid-S, oil-O, and water-W phases in food processing, it is necessary to find a new carrier of a delivery system.

The S/O/W technology is based on the reduction in interfacial tension between the O phase and S phase through lipid-soluble emulsifier, and the diffusion from the S phase to the O phase leads to the formation of suspension-like liquid. The mixed S/O phase and W phase form uniform S/O/W three-phase carrier microspheres through the mechanical action of a water-soluble emulsifier. As the carrier of food nutrients, S/O/W three-phase carrier microspheres have the advantages of low production cost and simplicity. The interaction of S, O, and W phases in S/O/W microspheres, the composition of W phase, and particle size distribution of microspheres determine the solubility, rheological properties, physicochemical stability, and digestibility of the carrier.

At present, there are few studies on the protection of nutrients by S/O/W technology. S/O/W was mainly focused on the fabrication of enzyme preparations and probiotics carriers. It showed that the S/O/W microspheres with superoxide dismutase, polyethylene glycol, and polylactic acid, and the superoxide dismutase microspheres exhibited suitable enzyme activity [9]. Spray-dried lactase to form a powder and then formed microspheres based on S/O/W technology to improve the activity of lactase, with the embedding rate over 75%. The stability of lactase was significantly improved compared with the traditional emulsion [10]. Meanwhile, the protection of NRRL B-30514 based on S/O/W technology was studied, and it was found that the formation of microspheres with powdered probiotics and beet pectin could effectively improve the environmental tolerance and digestive characteristics of probiotics [11]. The interaction between S, O, and W phases in S/O/W and the interaction between insoluble calcium and milk protein was reported that the surface of calcium salt particles could adsorb milk protein, and the absolute value of potential on the surface of calcium salt was increased obviously after adsorption [12].

Based on S/O/W technology, microspheres with specific structure distribution were constructed and formed to become a new carrier of a solid-phase nutrient delivery system [13]. Sodium caseinate (NaCas) is commonly used as an emulsifier in fabricating delivery systems. Because it can quickly adsorb at the oil-water interface in the emulsification process, reduce the interfacial tension and form a relatively thick interfacial layer, and prevent the flocculation and coalescence of newly formed droplets through electrostatic repulsive and steric effect, thus prolonging the stability of droplets in the emulsion [14]. Gillies’ team heated the cross-linked casein to form the emulsion with a dense network structure, the diffusion of droplets was slowed down, and the rheological properties of the emulsion were improved. Based on Van’t Hoff’s theory, the dissipative interaction of droplets was established, and the relationship between rheological properties and stability was established [15].

Despite the importance of delivery systems in the form of CaCO_3_ and S/O/W emulsions, few studies have been published. This is because to realize the three-phase homogenization of solid-S, oil-O, and water-W in the process of food processing and to solve the dispersion stability of insoluble calcium salts in liquid food is the key. Therefore, the S/O/W calcium-lipid microspheres were fabricated with CaCO_3_, the most commonly used calcium source as the S phase and soybean oil as O phase, and sodium caseinate as the W phase. Based on the high-speed shearing technique, the calcium-lipid microspheres were explored. The particle size distribution, zeta potential, and stability of the microspheres were explored. The stability and rheological mechanism of S/O/W calcium-lipid microspheres were investigated by combining the microstructure, shear rheological and microrheological Brownian trajectories, and tribological analysis. The research of S/O/W calcium-lipid microspheres emulsions can provide a theoretical and a technical basis for the delivery of calcium.

## 2. Materials and Methods

### 2.1. Materials

CaCO_3_ was bought from Shuang Teng Industrial Co., Ltd. (Zhengzhou, China). Sodium caseinate (Lot#C10185129) was acquired from Macklin Biochemical Co., Ltd. (Shanghai, China). Soybean oil was purchased from COFCO Co., Ltd. (Tianjin, China). Nile blue A and Nile red were obtained from Sigma-Aldrich Co. (St. Louis, MO, USA). All other chemicals were of analytical grade.

### 2.2. Preparation of S/O/W Calcium—Lipid Emulsions

Solution preparation: 2, 4, 6, 8, and 10 wt% NaCas solutions were prepared separately by mixing them with phosphate buffer (1.0 mM, pH 7.0). These solutions were stirred for at least 2 h in a water bath at 50 °C and then stored overnight at 4 °C to ensure full dissolution. The NaCas solutions were prepared to form the W phase.

S/O emulsions: CaCO_3_ and soybean oil at a ratio of 1:10 (*w*/*w*) using a magnetic stirring for 1 h to ensure the S phase suspended in the O phase, followed using a high-speed blender for 3 min (ULTRA TURRAX T25 digital, IKA, Staufen, Germany) with the speed of 15,000 rpm to form S/O emulsions.

S/O/W emulsions: S/O emulsions were mixed with NaCas solution at a ratio of (5%:95%, 10%:90%, 15%:85%, 20%:80%, 25%:75%, *w*/*w*), which was then using a high-speed blender for 5 min (ULTRA TURRAX T25 digital, IKA, Staufen, Germany) with the speed of 15,000 rpm to form S/O/W emulsions.

Ultra-pure water was used for all the experiments.

**Figure 1:**
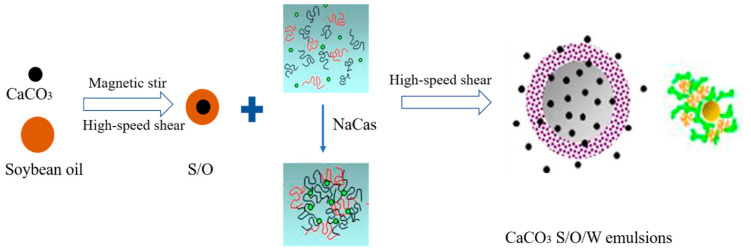
Schematic diagram of the preparation of S/O/W emulsion.

The scheme of the S/O/W emulsion system was shown in Figure 1.

### 2.3. Zeta Potential, Particle Size Distribution, and Physical Stability Measurements

The zeta potential of S/O/W emulsions was measured by Zetasizer Nano-ZS90 (Malvern Instruments, Worcestershire, UK). Emulsions were diluted with 1.0 mM phosphate buffer solution before the measurements, which was to avoid multiple scattering effects, and then injected the samples into a capillary cell equipped with two electrodes. The data were calculated from the measured electrophoretic mobility over 11 continuous readings according to the Smoluchowski theory after the samples were equilibrated for about 120 s. Each model emulsion was prepared at least in triplicate.

The particle size distribution of S/O/W emulsions was measured by a laser diffraction instrument (S3500 Microtrac Inc., Largo, FL, USA). The tri-laser system eliminates the influence of different wavelength light sources on the “connection point” of the scattering light distribution of particles and the errors of multiple Mie theory processing. All samples were measured at 25 °C. Measurements were performed in duplicate for each sample, and the results were expressed as average values. It is important to realize, as the samples were diluted, that the structure of S/O/W emulsions may change, and therefore the particle size distribution data should only be considered to provide a rough approximation of the true size to the extent of aggregation in the systems [16].

The physical stability of S/O/W emulsions was measured by the LUMiSizer (LUM GmbH, Berlin, Germany), which can analyze the occurrence of instability phenomena such as sedimentation, flocculation, or creaming. The instability index is used to determine the migration of particles in the emulsion by recording the changes in the distribution of transmittance in different positions of the sample under the irradiation of near-infrared light on the sample tube during the centrifugation process. The instability index can be obtained by further analysis and calculation of the original transmission curve by software, which can quantitatively represent the physical stability of the sample. At constant centrifugal speed, 12 different samples can be analyzed. The measurement parameters were as follows: sample injection volume 0.4 mL, rotational speed 2000 rpm, time interval 10 s, 200 spectral lines, test temperature 25 °C, and repeated measurement 3 times.

### 2.4. Viscosity Measurements

The shear viscosity of S/O/W emulsions was measured by continuous shear test using HAAKE rheometer (Mars IQ Air, Karlsruh, Germany). The measurement parameters were as follows: flat rotor type CC25 DIN, shear rate 2–200 s^−1^, test temperature 25 °C.

### 2.5. Microrheological Properties Analysis

The microrheometer is based on the theory of multi-speckle diffusion wave spectroscopy to analyze the microrheological properties of soft matter. By tracking the Brownian motion trajectory of the sample particles, the root means square displacement (MSD) curve is obtained to reflect the viscoelastic characteristics of the sample. The solid-liquid equilibrium value (SLB) is obtained by calculating the slope of the MSD platform region. The calculation equation of SLB is shown in Equation: SLB = MSD slope plateau. It characterizes the rheological characteristics such as structure, gel point, viscoelasticity of the emulsions, etc. The parameters were as follows: laser light source 650 nm, sample volume 20 mL, test temperature 25 °C.

### 2.6. Friction Coefficient Analysis

The friction coefficient of S/O/W emulsions was analyzed by using a DHR-1 tribo- rheometer. The emulsion was placed on the ball-disk surface and shears with the steel ball to simulate the friction process between the surface of the tongue and the roof of the mouth. The parameters are as follows: test pressure 1 N, test temperature 37 °C.

### 2.7. Microstructure Analysis

#### 2.7.1. Confocal Laser Scanning Microscopy Microstructure

The microstructure observation of S/O/W emulsions was visualized using a confocal laser scanning microscopy (CLSM) (FV3000, Olympus, Tokyo, Japan). The oil phase (Soybean oil) was pre-stained with Nile red. External protein suspension was stained with Nile blue A (2, 4, 6, 8, and 10 wt% NaCas) before emulsification. They were then kept overnight in the dark at 4 °C to completely dissolve the dye. Nile red uses a 488 nm laser excitation source, and Nile blue A uses a 635 nm laser excitation source. A 10× eyepiece and a 60× objective lens (oil immersion) were used to magnify the samples. The CLSM images were read and analyzed by the software of the instrument (FV10-ASW 4.1 Viewer, Olympus, Tokyo, Japan).

#### 2.7.2. Cryo-Scanning Electron Microscopy Microstructure

The cross-sectional and interfacial structure of S/O/W emulsions were observed using cryo-scanning electron microscopy (Cryo-SEM) (Helios NanoLab G3 UC, FEI, Hillsboro, OR, USA) based on a method reported previously to obtain more intuitive information [17]. Emulsion samples were immersed into liquid nitrogen and then transferred to a cryo-preparation chamber (PP3010T, Quorum Technologies, Lewes, U.K.) under vacuum. After freeze-fracturing and high vacuum sublimation to sublimation at −95 °C for 10 min to unbound water, and then the samples were sputter-coated with platinum and imaged using SEM. The observation was carried out at a distance between 3 and 5 mm with TLD detection at 2 kV.

### 2.8. Infrared Spectral Analysis

The infrared spectral analysis of CaCO_3_ (S phase), NaCas solution (W phase), O/W emulsions, and S/O/W emulsions were analyzed by using an IS10 FT-IR spectrometer. The W phase, O/W emulsions, and S/O/W emulsions were pre-frozen at −80 °C for 24 h and then moved to vacuum freeze-drying for 48 h to obtain the samples of powder. The parameters were as follows: wavenumber range of 400–4000 cm^−1^, the resolution of the spectrometer was 4 cm^−1^, the signal and emission ratio was 50,000:1, and the spectrometer scanned 64 times.

### 2.9. XRD Measurements

The XRD analysis of CaCO_3_ (S phase), NaCas solution (W phase), O/W emulsions, and S/O/W emulsions were analyzed by using BRUCKER D8 ADVANCE (Brooke, Germany). The O/W emulsions and S/O/W emulsions were pre-frozen at −80 °C for 24 h and then moved to vacuum freeze-drying for 48 h to obtain the samples of powder. The parameters were as follows: the spectral range was set to 5–50 degrees, the scanning rate is set to 2 degrees/min, the acceleration voltage is 40 kV, and the tube current is set to 40 mA.

### 2.10. Statistical Analysis

At least triplicate measurements were performed for each experiment. The results calculated from these triplicate measurements are presented as means ± standard deviations. Origin 8.5 software was used to perform the statistical analysis.

## 3. Results

### 3.1. Zeta Potential, Particle Size Distribution, and Physical Stability of CaCO_3_ S/O/W Emulsions

**Figure 2:**
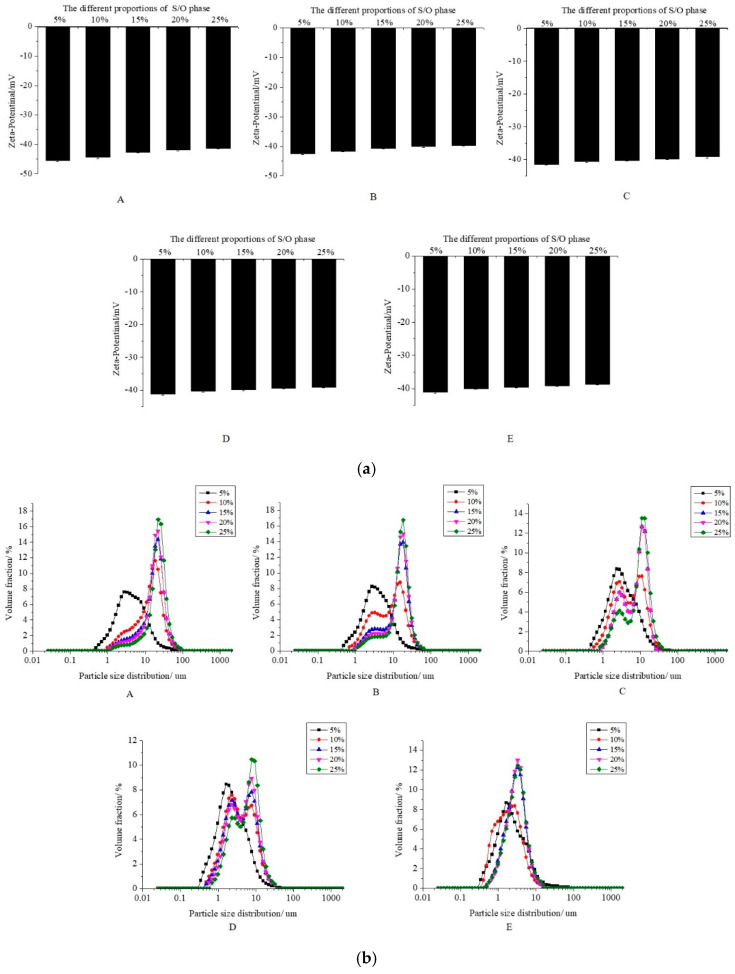

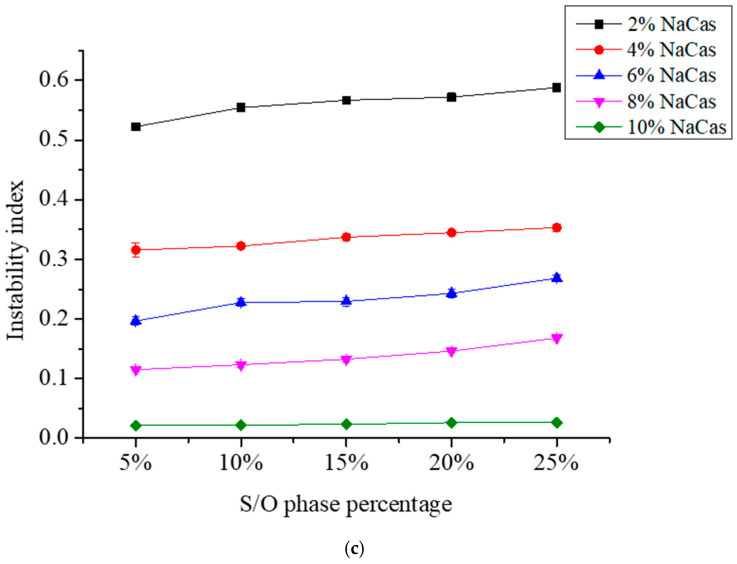
The zeta potential; (**a**) particle size distribution; (**b**) and instability index; (**c**) of S/O/W emulsions at different concentrations of NaCas and different proportions of S/O phase; (A, 2 wt% NaCas; B, 4 wt% NaCas; C, 6 wt% NaCas; D, 8 wt% NaCas; E, 10 wt% NaCas).

The zeta potential, particle size distribution, and physical stability of S/O/W emulsions at different concentrations of NaCas and different proportions of the S/O phase were shown in Figure 2.

#### 3.1.1. Zeta Potential of CaCO_3_ S/O/W Emulsions

The zeta potential of CaCO_3_ S/O/W emulsion was negative regardless of the different concentrations of NaCas or the different amounts of the S/O phase (Figure 2a). When the addition amount of S/O phase was constant, the zeta potential of emulsions were −43.17 ± 1.71 to −40.94 ± 1.17, −40.26 ± 0.86, −39.98 ± 0.82, and −39.71 ± 0.90 mV with increasing the content of NaCas (2–10 wt%), respectively. It was reported that NaCas had the pI at pH 4.6, where the net electrical charge of the protein was close to zero [18]. When the pH value of the system was higher than the pI of NaCas, the NaCas molecule was negatively charged. It could be explained by the fact that NaCas in the emulsion structure is placed at the interface of the oil droplets and confers negative values of zeta potential to the emulsion interface. The electrostatic repulsion between the NaCas was strong enough to overcome the attractive forces such as van der Waals, hydrophobic interaction, and depletion as the NaCas was lower, and the zeta potential was increased when the NaCas was higher. The zeta potential of CaCO_3_ S/O/W emulsions were negatively changing from −35.61 ± 1.22 to −34.33 ± 1.34, −33.37 ± 1.83, −32.18 ± 1.13, and −30.59 ± 1.52 mV as the addition amount of S/O phase increased. This was because the NaCas decreased as the S/O phase improved, and the absolute value of zeta potential of CaCO_3_ S/O/W emulsions decreased. The electrostatic repulsion among the oil droplets was ensured by the zeta potential that was strong enough to avoid droplets aggregation. This was consistent with the particle size distribution in Figure 2b.

#### 3.1.2. Particle Size Distribution of CaCO_3_ S/O/W Emulsions

The particle size distribution of CaCO_3_ S/O/W emulsions at different concentrations of NaCas and different proportions of the S/O phase were shown in Figure 2b. When the addition of the S/O phase is constant, the particle size decreased gradually with the increase in NaCas concentration (2–10 wt%), and the distribution range of particle size was narrow. It could be explained that the total interface area stabilized by NaCas at low concentration was small, which led to the agglomeration of droplets. With the increase in NaCas concentration, the total interface area stabilized by NaCas was larger, and then the particle size of the emulsion decreased. The CaCO_3_ S/O/W emulsion particle size increased as the proportion of the S/O phase increased. This was because when the S/O phase was lower and the W phase was higher, the emulsifying effect was better, the emulsion particle size was smaller, and the distribution was more uniform. With the increase in the S/O phase, the emulsification efficiency decreased, which led to the coalescence of droplets and the increase in emulsion particle size.

#### 3.1.3. Physical Stability of CaCO_3_ S/O/W Emulsions

The effects of NaCas and the additive amount of the S/O phase on the physical stability of CaCO_3_ S/O/W emulsions were shown in Figure 2c. The instability index can be obtained by further analysis and calculation of the original transmission curve by software, which can quantitatively represent the physical stability of the emulsion. The lower the instability index, the better the stability of the emulsion.

Figure 2c shows that when the S/O phase was in a certain amount, CaCO_3_ S/O/W emulsions instability index reduces gradually in lowest (0.01) as the NaCas increased up to 10 wt%, it was because the higher NaCas contributed to the viscosity of the emulsions, which would cause the stability of the emulsion improved. With a constant concentration of NaCas, the S/O/W emulsion instability index rose as the increase in S/O phase amount; this is because the S/O phase increase as well as the W external water phase content decreases, the emulsifying effect decreases. The emulsifier couldn’t cover the particles completely makes the oil droplets easy to aggregate. Therefore, the O phase immediately aggregates and precipitates after the emulsification process, which would cause the emulsion to stratify and precipitate and making it unable to form the emulsion. The experimental results show that the S/O/W emulsion has the best stability when the S/O phase addition is 5%. With the increase in the S/O phase addition, the S/O/W emulsion has worse stability [19,20]. In the emulsification process, NaCas can adsorb at the oil-water interface, reduce the interfacial tension and form a relatively thick interfacial layer, and prevent the flocculation and coalescence of newly formed droplets through electrostatic repulsion and steric effect, thus prolongs the stability of droplets in the emulsion.

### 3.2. Rheological Properties of CaCO_3_ S/O/W Emulsions

**Figure 3:**
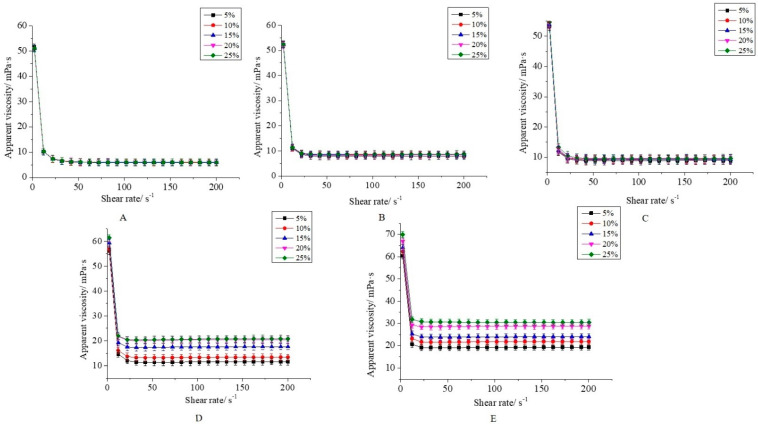
Apparent viscosity of S/O/W emulsions at different concentrations of NaCas and different proportions of S/O phase determined by rotation rheometer. (**A**) 2 wt% NaCas; (**B**) 4 wt% NaCas; (**C**) 6 wt% NaCas; (**D**) 8 wt% NaCas; (**E**) 10 wt% NaCas).

Increasing the viscosity is an important way to improve the stability of the CaCO_3_ S/O/W emulsions. The viscosity of S/O/W emulsions with different concentrations of NaCas and various amounts of S/O phase at the shear rate range of 2–200 s^−1^ were shown in Figure 3. The viscosity of S/O/W emulsions decreased significantly and then became stable as the shear rate increased, which indicated that the S/O/W emulsions had shear-thinning fluid characteristics. This was because the higher shear rate caused the honeycomb three-dimensional space network structure of S/O/W emulsion particles destroyed to a certain extent so that the emulsion particles flowed in a certain direction and the flow resistance decreased and then apparent viscosity lower [21].

Generally, the stability in apparent viscosity vs. shear rate always occurred from 25 to 200 s^−1^, and that when the shear rate increased, particles would be orientated along the flow direction. The viscosity of S/O/W emulsions increased slightly with the increase in NaCas. When the shear rate was 200 s^−1^, the viscosity increased from 7.31 ± 0.130 (2 wt% NaCas) to 27.80 ± 0.131 mPa·s (10 wt% NaCas). NaCas not only played a role in the thickening of the particles but also constitute the structure of the honeycomb three-dimensional space network, and thus improving the stability of S/O/W emulsion particles. S/O phase content of S/O/W emulsion particles had little impact on viscosity, with the increase in S/O phase content and lower NaCas, S/O/W emulsion particle viscosity was low, and showed clear non-newtonian flow (shear thinning) curve, the viscosity with the increase in shear rate was significantly lower, it mainly reflected the emulsion droplets deflocculation [22,23]; with the increased of S/O phase content and higher NaCas, the viscosity increased as the increase in shear rate significantly but then reduced after the phenomenon of a slight increase, this was due to the O phase itself has a certain viscosity, when the O phase increased the emulsion viscosity increased [24]. The above results indicated that the concentration of NaCas had a greater effect on the viscosity of S/O/W emulsion particles than the additional amount of the S/O phase.

### 3.3. Microrheological Property of CaCO_3_ S/O/W Emulsions

**Figure 4:**
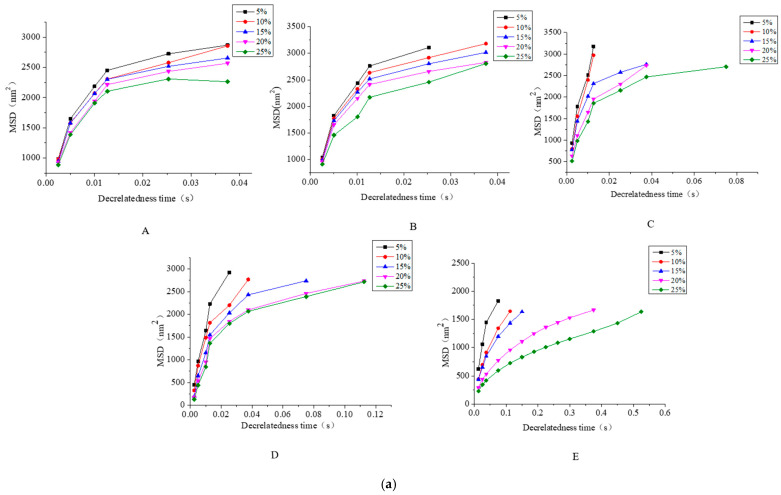

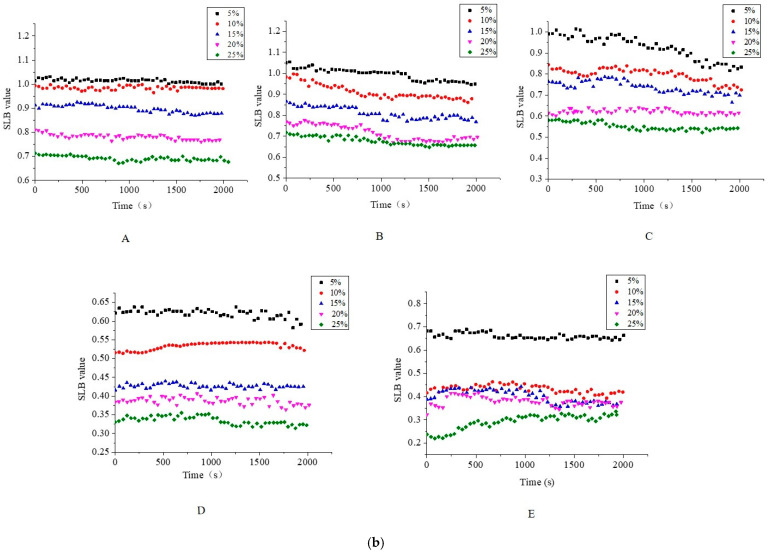
The microrheological properties of S/O/W emulsions at different concentrations of NaCas and different proportions of S/O phase determined by an optical microrheometer (**a**) MSD (**b**) SLB (A, 2 wt% NaCas; B, 4 wt% NaCas; C, 6 wt% NaCas; D, 8 wt% NaCas; E, 10 wt% NaCas).

Rheolaser Lab is an optical microrheological analysis instrument, which can be used to analyze the viscoelastic properties of emulsion in micron scale. By measuring the displacement of the particles in the emulsion due to thermal energy (Brownian motion), their own viscoelastic properties can be obtained. The trajectory of the particles can reflect the structure of the material. According to the root mean square curve of particle displacement, different parameters can be obtained, such as the root mean square displacement (MSD) and the solid-liquid equilibrium value (SLB) of the sample particles. Measurements are made completely at rest with no mechanical shear force, and the original viscoelastic results are obtained completely without any modification. The effects of different concentrations of NaCas and the amount of S/O phase addition on the micro-rheology of CaCO_3_ S/O/W emulsions were shown in Figure 4.

Figure 4a shows the effects of S/O/W emulsion of different NaCas concentrations and different S/O phase additions on the microrheological root mean square displacement (MSD). When the amount of the S/O phase was constant, the MSD curve of the S/O/W emulsion gradually increased linearly with increasing the content of NaCas, and it belonged to viscous fluid. MSD decreased significantly with the increase in sodium caseinate concentration, and the final MSD value was the minimum when the concentration of NaCas was 10 wt%, indicating that a complete and stable three-dimensional spatial network structure had been formed at this time, thus limiting the Brownian motion space of the particles and improving the stability. In addition, the particle viscosity of S/O/W emulsion increased significantly with the increase in NaCas concentration, which was also consistent with the results of shear rheology (Figure 3). The MSD curve of S/O/W emulsion prepared with different S/O phase addition increased linearly and showed more vicious behavior, and it belonged to viscous fluid. When the amount of the S/O phase was higher, the larger MSD would be generated, which indicated that the Brownian motion region of particles was larger and the stability was poorer.

Figure 4b shows the solid-liquid equilibrium value (SLB) of the emulsion with different concentrations of NaCas and different S/O phase addition amounts of vegetation, which is used to evaluate the solid-liquid state of the sample. When the SLB < 0.5, it means that the droplet motion shows similar elastic behavior; when SLB = 0.5, solid characteristics and liquid characteristics of the sample are equal; when the SLB > 0.5, it shows more liquid (or viscous) behavior, and SLB greater than 1 indicates the occurrence of sediment. The lower the SLB value, the more solid characteristics of the samples were displayed. The lower the SLB value of emulsion, the more motion restricted of the emulsion droplets and the lower motion rate. In addition, the viscoelastic properties of the emulsion can be explained by the value of solid-liquid equilibrium (SLB) in micro-rheology. As shown in Figure 4b, SLB value gradually decreased with the increase in S/O phase addition in the S/O/W emulsion, indicating that the rheological behavior of the emulsion changed from vicious behavior to elastic behavior. With the increase in NaCas concentration, the SLB value increased. At low NaCas concentration, the elastic behavior of the emulsion was dominant, while at high NaCas concentration, the viscosity behavior of the emulsion was dominant. At the same time, SLB results could also reflect the stability of S/O/W emulsions, which was consistent with Figure 3C.

### 3.4. Tribological Analysis of CaCO_3_ S/O/W Emulsions

The lubrication behavior of S/O/W emulsions can be expressed using the Stribeck friction curve. In the Stribeck curve model, the curve is divided into boundary area, mixing area, and hydrodynamic area according to different friction behaviors between two contact surfaces [25]. In the boundary region, the friction between the two contact surfaces is almost not affected by sliding speed or sample viscosity but is mainly affected by the ability of the sample to adsorb and form a lubricating film between the contact surfaces. The friction in this region is constant and mainly comes from the solid-solid friction between the contact surfaces. With the further increase in the shear velocity, the friction curve enters the mixing zone, forming a lubrication film that can reduce the friction, and the solid-solid friction weakens. The decrease in the friction depends on the sample particle size, which promotes the fluid entrainment, and the friction decreases with the increase in the velocity. At higher velocities, the friction curve enters the hydrodynamic zone, and the lubrication film is fully formed. At this time, the friction is completely dependent on the viscosity of the sample and increases with the increase in velocity [26].

**Figure 5:**
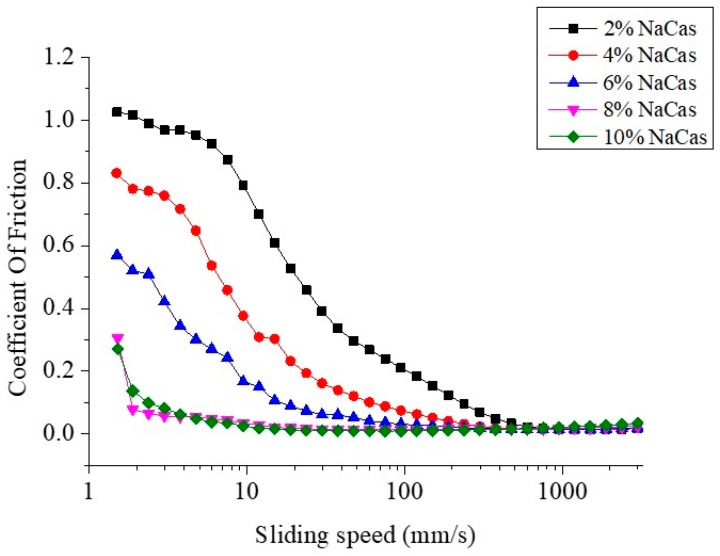
Friction coefficient of S/O/W emulsions at different concentrations of NaCas and 5% proportions of the S/O phase was measured by a tribometer at 37 °C simulated oral rejection.

Figure 5 shows the change of coefficient friction of S/O/W emulsion prepared with different concentrations of NaCas. When the NaCas was 2 wt%, due to the low protein particle concentration and the free flow of water molecules was more when in contact with the probe of friction contact, the S/O/W emulsions showed a trend of decrease under low shear stress. When the concentration of NaCas increased, more protein particles gathered around the contact area, under the action of shear force, the protein particles gather between the contact surfaces, forming a “ball bearing” lubrication effect, further reduced the friction coefficient, and with the excessive accumulation of protein particles, showed a blocking effect. When the shear rate further increased, the combined influence factors made the whole showed the characteristics of shear thinning, and the overall trend decreased [27]. Studies have found that differences in tribological behavior could also be explained based on the difference in the rheology of the system. Rheological results (Figure 3) reflected that the S/O/W emulsion had a lower viscosity distribution, which was mainly characterized by boundary lubrication. There was a platform in the S/O/W emulsion friction coefficient at low speeds between 1 and 4 mm/s, which indicates boundary lubrication. At speeds above 4 mm/s, the friction coefficient decreased with increasing speed, indicative of the mixed lubrication. The relationship between the rheology of S/O/W emulsion and tribology is a hot topic in this study. The effect of oil content and W phase-type on the tribological behavior of the emulsion system needs to be further studied.

### 3.5. Microstructure of CaCO_3_ S/O/W Emulsions

#### 3.5.1. Confocal Laser Scanning Microscopy

**Figure 6:**
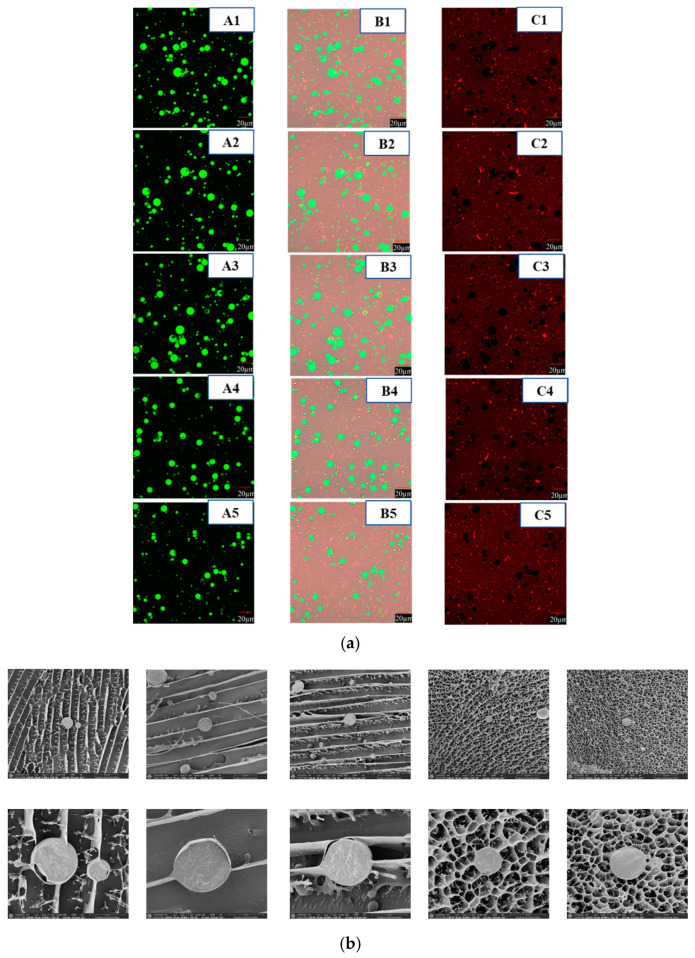
The CLSM microstructure (**a**) and Cryo-SEM imaging; (**b**) of S/O/W emulsions at different concentrations of NaCas and 5% proportions of S/O phase. ((**a**): A, O phase was stained with Nile red, excitation at 488 nm; B, W phase was stained with Nile blue, excitation at 635 nm (ii); C, combined image). Scale bar: 20 μm; (**b**): A, 5000×; B, 20,000× (1–5: 2, 4, 6, 8, and 10 wt% NaCas, respectively)).

The microstructure of CaCO_3_ S/O/W emulsion microparticles was observed using a confocal laser scanning microscopy (CLSM) (FV3000, Olympus, Tokyo, Japan). In the CLSM images, soybean oil droplets appeared green, NaCas molecules showed red, and CaCO_3_ molecules (without dye) exhibited black [17]. The combined oil phase and protein phase were shown in Figure 6a.

It could be observed that the particle size of S/O/W emulsions was comparatively small and uniformly dispersed as the NaCas concentrations increased, which was consistent with the results in Figure 2b. When the concentration of NaCas was lower, the particle size distribution range of S/O/W emulsions was wide, the particle zeta potential was low, and the emulsions were instability. As the concentration of NaCas increased, the electrostatic repulsive force increased, and the emulsion particle distribution became uniform, and the stability was enhanced, which was consistent with the results in Figure 2c. With the increase in NaCas, the embedding effect of S/O/W emulsions on CaCO_3_ was slightly enhanced, which was due to the overall increase in the stability of the emulsion and the decrease in calcium precipitation. Because CaCO_3_ has strong hydrophilicity and its hydrophilicity is stronger than lipophilicity, the microstructure of CaCO_3_ S/O/W emulsions formed generally has a calcium embedding effect close to the outer aqueous phase [28], that is at the edge of the emulsion microspheres. Some studies used confocal laser microscopy to monitor the embedding of probiotics NRRL B-30514 in S/O/W emulsion, but the probiotics were not accurately located [11]. In this study, the experimental results of laser confocal microscope could be observed and accurately located the embedding of CaCO_3_ in S/O/W emulsion, which proved that CLSM can monitor the encapsulation of calcium in liquid food, providing new ideas and new methods for future experimental research.

#### 3.5.2. Cryo-Scanning Electron Microscopy

The cross-section structure and the attachment of emulsions particles at the external interface were revealed by cryo-scanning electron microscopy (Cryo-SEM), which were shown in Figure 6b. The dark zones were places previously occupied by the water before sublimation. It was found that the CaCO_3_ S/O/W emulsions of lower NaCas showed flake structure with some filaments while exhibited lots of bigger droplets connected through filaments; the S/O/W emulsions of higher NaCas formed a honeycomb three-dimensional (3-D) spatial network structure, in which the emulsion droplets were uniformly distributed. The formation of a box-shaped structure provided steric hindrance and a highly charged layer to help emulsions against isoelectric flocculation. The Cryo-SEM microstructures were corresponding to the consequences of particle size, zeta potential and physical stability measurements. Cryo-SEM microstructure further proved that the increase in NaCas could promote the formation of honeycomb three-dimensional spatial network structure of S/O/W emulsion particles and improved the physical stability [29]. The construction of S/O/W calcium-lipid microspheres was proved by the core-shell structure observed by Cryo-SEM and the calcium embedding condition monitored by CLSM (Figure 6). Some studies have prepared micro-powdered nanoparticles calcium carbonate into gelated sodium alginate nanospheres [29] or S/O/W suspension and then carried out spray drying [3]. In this study, micron-sized calcium carbonate was directly embedded in the S/O/W emulsion system. The system innovation was very important, and it provided a theoretical basis for solving the dispersion stability of insoluble calcium salt in liquid food.

### 3.6. Infrared Spectra Analysis of CaCO_3_ S/O/W Emulsions

**Figure 7:**
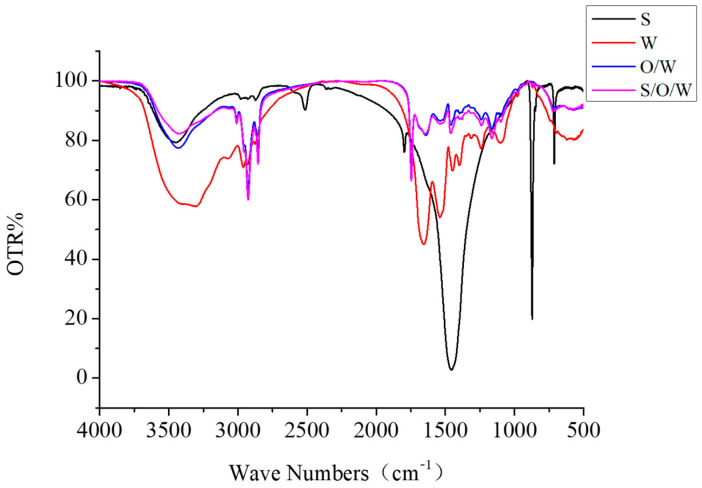
The infrared spectrum results of S phase, W phase, O/W emulsions, and S/O/W emulsions.

The infrared spectrum results of S phase, W phase, O/W emulsions, and S/O/W emulsions were shown in Figure 7. The characteristic absorption peaks of CaCO_3_ (S phase) appeared at 3446.12, 2513.39, 1796.99, 1456.26, 872.54, and 712.67 cm^−1^, among which the C-O stretching vibration peak appeared at 1796.99 cm^−1^. There is a strong absorption peak at 1456.26 cm^−1^, which is the characteristic absorption peak of calcium carbonate powder and represents the asymmetric stretching vibration of the C-O bond. The absorption peaks of 872.54 and 712.67 cm^−1^ are V2 and V1, respectively. V2 peak is strong and sharp, while V1 peak is sharp but not as strong as V2. They are related to the bending vibration of the C-O bond, and CO_3_^2−^ out-of-plane deformation vibration peak appears at 872.54 cm^−1^. The in-plane deformation vibration peak of CO_3_^2−^ appears at 712.67 cm^−1^ [30,31,32].

The characteristic absorption peaks of NaCas (W phase) appeared at 3306.28 cm^−1^, 2961.05 cm^−1^, 1655.03 cm^−1^, 1539.57 cm^−1^, 1448.47 cm^−1^, 1398.40 cm^−1^, 1241.17 cm^−1^, 1102.62 cm^−1^, and 573.57 cm^−1^, among which the stretching vibration of the O-H bond appeared at 3306.28 cm^−1^; the stretching vibration peak of C-H bond appeared at 2961.05 cm^−1^; 1600~1700 cm^−1^ is the absorption peak of protein amide I band (C = O stretching vibration); 1400–1550 cm^−1^ is the amide II band (NH deformation vibration, CN stretching vibration band), and 1200–1350 cm^−1^ is the amide band (N-H in-plane bending vibration and C-H stretching vibration). According to the literature, the secondary structure distribution of the amide band is as follows: 1640–1640 cm^−1^ is β-folding, 1650–1670 cm^−1^ is α-helix, 1640–1650 cm^−1^ is the random coil, and 1680–1685 cm^−1^ is β-corner structure [33,34,35].

The characteristic absorption peaks of O/W emulsions appeared at 3431.30, 2926.08, 2854.82, 1746.35, 1644.02, 1540.28, 1458.03, 1240.35, 1164.11, and 576.47 cm^−1^. Compared with the W phase, the absorption intensity of the absorption peak of the O/W emulsion weakened, the wavenumber increased, and the blue shift occurred, which may be caused by the decrease in the size of emulsion particles [36]. The characteristic absorption peaks of O/W emulsions appeared at 3425.10, 3009.33, 2925.79, 2854.55, 1746.45, 1645.34, 1538.64, 1457.02, 1238.63, 1162.79, and 587.71 cm^−1^. The presence of CaCO_3_ changed the vibration of micro-groups in the emulsion, and the absorption peak intensity of S/O/W increased compared with that of O/W, but there was no red shift or blue shift. It may be that the two do not form covalent bonds but combine with a weak force.

### 3.7. XRD Analysis of CaCO_3_ S/O/W Emulsions

**Figure 8:**
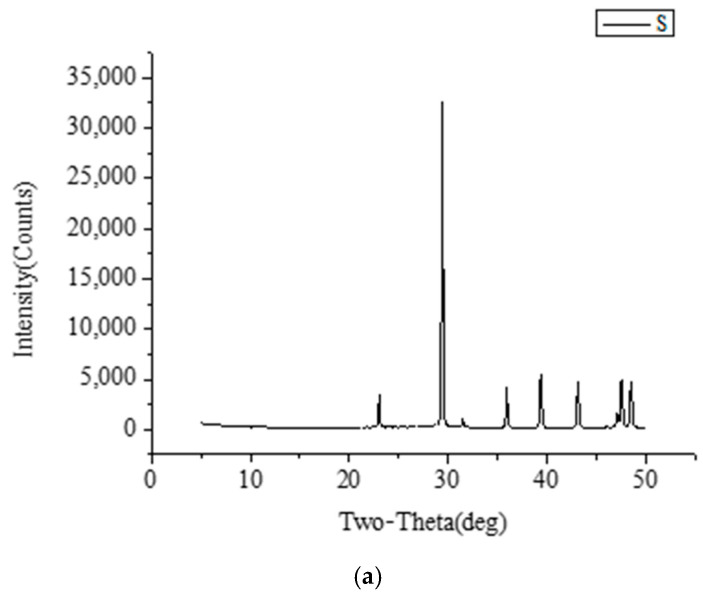

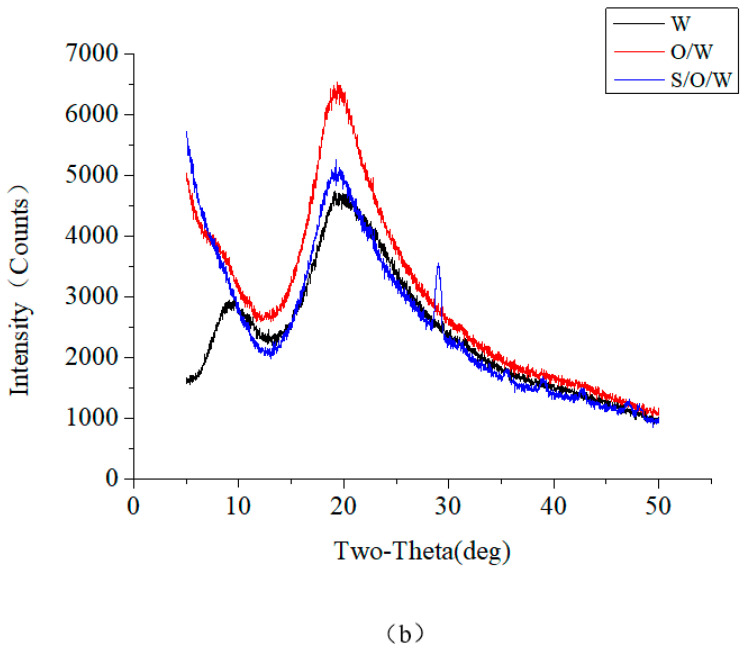
The X-ray results of (**a**) S phase, (**b**) W phase, O/W emulsions, and S/O/W emulsions.

The XRD results of the S phase, W phase, O/W emulsions, and S/O/W emulsions were shown in Figure 8. XRD analysis proved that CaCO_3_ (S phase) formed the common calcite structure in the absence of matrix with the diffraction peak of calcite structure: 23 (012), 29 (104), 36 (110), 39 (113), 43 (202), 47.5 (018), and 48.5 (118) [37]. The diffraction peaks of NaCas (W phase) with XRD analysis were 10 and 20; the diffraction peak of O/W emulsions with XRD analysis was 20; the diffraction peak of S/O/W emulsions with XRD analysis was 20, 29, 36, 39, 43, 47.5, 48.5, which was the CaCO_3_ with the matrix. The results showed that when the CaCO_3_ was embedded in the S/O/W emulsion system, the characteristic diffraction peak in the XRD image was calcite crystal. The characteristic peak was more sharp compared with the blank CaCO_3_. When the emulsion system was added, the width of the peak becomes larger because the O/W phase was involved in the crystallization process, and the crystal formed was defective, which was not as perfect as the blank calcium carbonate crystal, resulting in lattice distortion [38].

## 4. Conclusions

The present study showed that when the NaCas was 10%, it was more feasible to encapsulate CaCO_3_ with S/O/W emulsion because of the reduced particle size and improved stability against aggregation and coalescence. Meanwhile, the microstructure indicated that the S/O/W system was successfully constructed. When the S/O phase was 5%, the S/O/W emulsion has better stability and rheological characteristics due to the presence of free NaCas. By studying the dispersion and stability of insoluble calcium salts in liquid food, the theoretical foundation is laid for the design of a three-phase (S, O, W) matrix transfer system, which can solve the technical problems of solid-phase nutrient components processing and has a broad application prospect in the food and pharmaceutical industry.
